# Expression of the Na^+^/l^- ^symporter (NIS) is markedly decreased or absent in gastric cancer and intestinal metaplastic mucosa of Barrett esophagus

**DOI:** 10.1186/1471-2407-7-5

**Published:** 2007-01-10

**Authors:** Áron Altorjay, Orsolya Dohán, Anna Szilágyi, Monika Paroder, Irene L Wapnir, Nancy Carrasco

**Affiliations:** 1 Department of Surgery, St. George University Teaching Hospital H-8000 Székesfehérvár, Hungary; 2 Department of Molecular Pharmacology, Albert Einstein College of Medicine, Bronx, NY 10461, USA; 3 Department of Pathology, St. George University Teaching Hospital H-8000 Székesfehérvár, Hungary; 4 Department of Surgery, Stanford University School of Medicine, Stanford, California 94305-5655, USA

## Abstract

**Background:**

The sodium/iodide symporter (NIS) is a plasma membrane glycoprotein that mediates iodide (I^-^) transport in the thyroid, lactating breast, salivary glands, and stomach. Whereas NIS expression and regulation have been extensively investigated in healthy and neoplastic thyroid and breast tissues, little is known about NIS expression and function along the healthy and diseased gastrointestinal tract.

**Methods:**

Thus, we investigated NIS expression by immunohistochemical analysis in 155 gastrointestinal tissue samples and by immunoblot analysis in 17 gastric tumors from 83 patients.

**Results:**

Regarding the healthy Gl tract, we observed NIS expression exclusively in the basolateral region of the gastric mucin-producing epithelial cells. In gastritis, positive NIS staining was observed in these cells both in the presence and absence of *Helicobacter pylori*. Significantly, NIS expression was absent in gastric cancer, independently of its histological type. Only focal faint NIS expression was detected in the direct vicinity of gastric tumors, i.e., in the histologically intact mucosa, the expression becoming gradually stronger and linear farther away from the tumor. Barrett mucosa with junctional and fundic-type columnar metaplasia displayed positive NIS staining, whereas Barrett mucosa with intestinal metaplasia was negative. NIS staining was also absent in intestinalized gastric polyps.

**Conclusion:**

That NIS expression is markedly decreased or absent in case of intestinalization or malignant transformation of the gastric mucosa suggests that NIS may prove to be a significant tumor marker in the diagnosis and prognosis of gastric malignancies and also precancerous lesions such as Barrett mucosa, thus extending the medical significance of NIS beyond thyroid disease.

## Background

**Figure 1:**
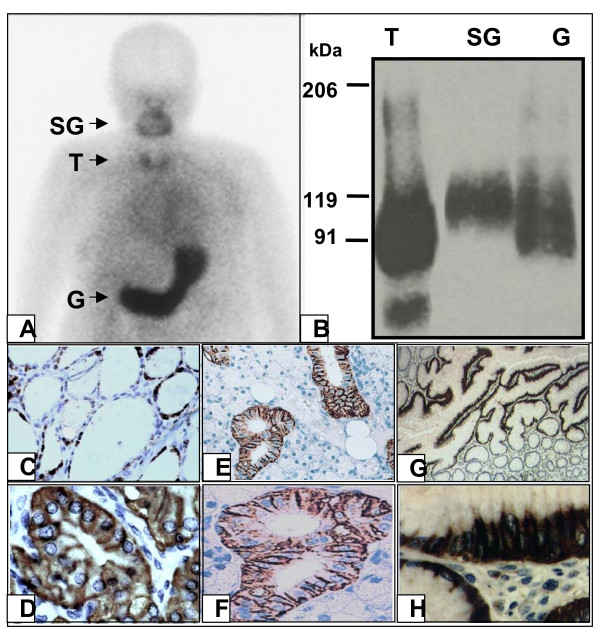
**NIS expression in different tissues**. **A**: Radioiodide accumulation in NIS-expressing human tissues (SG: salivary glands, T: thyroid, G: stomach) 2 hours after ^99m^Tc-pertechnetate administration (5 mCi). B: Immunoblot analyses of human NIS expression in a Graves' thyroid (T), normal salivary glands (SG), and normal gastric mucosa (G). Total protein (50 μg) was electrophoresed into each lane; the nitrocellulose membrane was probed with 3 nM affinity-purified anti-human-NIS Ab as described in Materials and Methods. C-H: Immunohistochemical analyses of NIS expression in human iodide-concentrating tissues. C: Normal thyroid (original magnification: 400 ×), D: Graves' thyroid, strong basolateral NIS staining of the follicular epithelial cells (original magnification: 1,000 ×), E: Salivary gland (original magnification: 400 ×), F: Basolateral NIS staining in the salivary ductal cells (original magnification: 1,000 ×), G: Gastric mucosa (original magnification: 400 ×), H: Basolateral NIS staining of the gastric mucin-secreting cells (original magnification: 1,000 ×).

Iodide (I^-^) is an essential constituent of the thyroid hormones triiodothyronine (T_3_) and tetraiodothyronine (T_4_). These hormones are vital for the normal development and maturation of the central nervous system in the newborn and for multiple metabolic functions in the adult. I^- ^metabolism in humans appears to have adapted to provide sufficient I^- ^for normal thyroid function in the face of the environmental scarcity of I^-^. A cornerstone of I^- ^metabolism is active I^- ^uptake in the thyroid, a process mediated by the sodium/iodide symporter (NIS)[1,2]. NIS is an integral plasma membrane glycoprotein located in the basolateral membrane of the thyroid follicular cells[3]. Although NIS-mediated active I^- ^uptake has long been viewed as a distinctly thyroidal phenomenon, it is now clear that active I^- ^transport observed in extrathyroidal tissues such as salivary glands, lactating mammary gland, gastric mucosa, and placenta is also mediated by NIS [3-8]. The NIS cDNA cloned from these tissues is identical to thyroid NIS[5]. Indeed, deglycosylation with N-glycosidase F or methionine-specific CNBr cleavage of thyroid, stomach, and mammary gland NIS proteins has indicated that NIS is the same protein in each of these tissues[6]. NIS-mediated radioiodide uptake in the stomach and salivary glands is routinely observed in radioiodide/^99m^TcO_4 _^-^whole-body scintiscans (Fig 1A) [9].

The supply of I^- ^for thyroid hormone biosynthesis is governed by dietary I^- ^intake, I^- ^absorption, and thyroidal I^- ^uptake. I^- ^is presumed to be absorbed in the small intestine, but neither the anatomical location of its absorption nor its mechanism has been identified. Interestingly, gastric NIS mediates the active transport of I^- ^from the bloodstream to the gastric lumen, i.e., the active *secretion *of I^- ^into the gastric juice. Secreted I^- ^is then recirculated into the bloodstream when it is absorbed, along with newly ingested dietary I^-^, in the small intestine. I^- ^is ultimately excreted mainly by the kidneys. The role of secreted I^- ^in the gastric juice is unknown, as is the function of NIS-mediated I^- ^secretion to the saliva in the salivary glands. By contrast, the functional role of NIS-mediated I^- ^translocation in the lactating mammary gland is crucial and very clear: the process results in I^- ^secretion to the milk, thus supplying the anion to the breast-fed newborn for his/her own thyroid hormone biosynthesis[6].

Data on NIS expression and function in regions of the gastrointestinal tract other than the stomach are still scant and somewhat controversial. The presence of the NIS transcript, as detected by RT-PCR, has been reported in both the colon[10] and the small intestine[11]. However, other investigators have been unable to amplify the NIS transcript in either of these two tissues[5,12,13]. By immunoshistochemistry, Spitzweg *et al*[5], Lacroix *et al *[13], and Wapnir *et al *[14] have observed some NIS protein expression in the colon; in contrast, Vayre *et al *[15] observed it only in the rectum but not in the rest of the colon. None of the investigations carried out to date have shown NIS expression in the esophagus[3]. Findings on NIS expression in gastrointestinal tumors have also been limited. Gastric carcinoma, unlike normal mucosa, has generally been reported to exhibit no I^- ^or pertechnetate (^99m^TcO_4 _^-^)accumulation [16-18] (pertechnetate is an anion with the same size and charge as I^- ^and is similarly transported by NIS), with the sole exception of Wu *et al *[19], who reported radioiodide uptake in gastric adenocarcinoma. In addition, in the 1960's and 70's, radioiodide or ^99m^TcO_4 _^-^gastric scintigraphy was studied as a possible method for the diagnosis of gastric neoplasia, based on the finding that malignant gastric tissue failed to transport I^-^or ^99m^TcO_4 _^-^into the gastric juice [16-18]. Furthermore, during the same decade, Berquist *et al *used ^99m^TcO_4 _^- ^scintigraphy to establish the diagnosis of Barrett esophagus, based on the characteristic replacement of the distal esophageal mucosa by the ^99m^TcO_4 _^-^concentrating gastric columnar epithelium[20,21]. These observations suggest not only that NIS expression may be impaired as a result of malignant transformation, but also that the determination of NIS expression and function may be of diagnostic value in gastroesophageal disease.

Consistent with the above concept is our recent report of decreased or absent NIS expression in 27 gastric adenocarcinomas studied by high-density tissue microarrays[14]. Whereas tissue microarrays are optimal for high-throughput screening, the sampling with tissue cores is limited Thus, to expand our findings and more thoroughly examine the issue of NIS expression in gastroesophageal cancer and its possible diagnostic value, we have analyzed NIS expression in normal and malignant gastrointestinal tissue samples from 83 patients. Samples were obtained during surgical resection or endoscopic examination and were analyzed by immunohistochemistry on conventional tissue sections, given that this technique offers the advantage (over RT-PCR) of determining expression and cellular localization of the NIS protein instead of the NIS transcript[22]. We also assessed NIS expression by immunoblot analysis to ascertain the specificity of the observed immunoreactivity.

## Methods

Snap frozen tissue samples were obtained for immunoblot analysis from 17 patients undergoing resections for gastric tumors (3 MALT and 14 adenocarcinomas from 5 female and 12 male patients; average age: 62). Corresponding normal peritumoral tissues were also collected.

We studied by immunohistochemistry tissue samples obtained from the gastrointestinal tracts of 66 patients (average age: 54; male/female ratio: 37/29). In the case of 20 cancer patients, samples were taken from tumors and their neighboring areas (tumor, tumor margin, and 1 cm and more than 3 cm from the tumor margin) immediately after resection in the operating theatre; the remaining 46 (66–20) were biopsies. Tissue samples from small and large intestines were obtained from all segments of the intestinal tract (jejunum, ileum, and right and left colon). Permission for the investigation was obtained from the local Ethical Committee of the St. George University Teaching Hospital Székesfehérvár, Hungary.

### Immunoblot

Tissue samples were blended for 1 min with a polytron homogenizer (Brinkman Instruments, Westbury, New York) and homogenized with a stirrer type glass-Teflon homogenizer (Caframo-Wiarton, Ontario, Canada) in a buffer containing 250 mM sucrose, 1 mM EDTA, 10 mM Hepes (pH 7.5), and protease inhibitors (90 μg/ml aprotinin, 4 μg/ml leupeptin, 0.8 mM phenylmethanesulfonyl fluoride). Membrane fractions were prepared as described[23]. PAGE and electroblotting to nitrocellulose were performed as previously described[23]. All samples were diluted 1:2 with sample buffer and heated at 37°C for 30 min prior to electrophoresis. Immunoblot analysis was also carried out as described[23], with affinity-purified anti-human-NIS (-hNIS) Ab[6,14] (1 μg/μl) at a 1:2,000 dilution, and a 1:2,000 dilution of a horseradish-peroxidase-linked donkey anti-rabbit IgG (Amersham). Both incubations were performed for 1 h. Proteins were visualized by the enhanced chemiluminescence (ECL) Western blot detection system (Amersham).

### Immunohistochemistry

All gastrointestinal tissue sections (3 μm) were deparaffinated and rehydrated. All slides were subjected to antigen retrieval by means of a 10%-citrate buffer. Washes were done with TBST [0.3 M NaCI, 0.1% Tween 20, 0.05 M Tris-HCI (pH 7.6)] for 5 min. Endogenous peroxide activity was blocked with 5% H_2_O_2 _for 15 min. Endogenous biotin activity was blocked with the DAKO Biotin Blocking System (Carpinteria, CA). Slides were incubated for 1 h with the affinity-purified polyclonal anti-human NIS antibody generated against the last 16 amino acids of the carboxy-terminal end of the protein[22]. The initial concentration of the Abs was 1 μg/μl; they were diluted in the blocking solution 1:6,000. The Catalyzed Signal Amplification kit (DAKO, Carpinteria, CA) was used for the remainder of the procedure according to the supplier's instructions. Salivary parotid gland was used as a positive control and mesenteric lymph node as a negative control. All slides were counterstained with hematoxylin.

## Results

**Figure 2:**
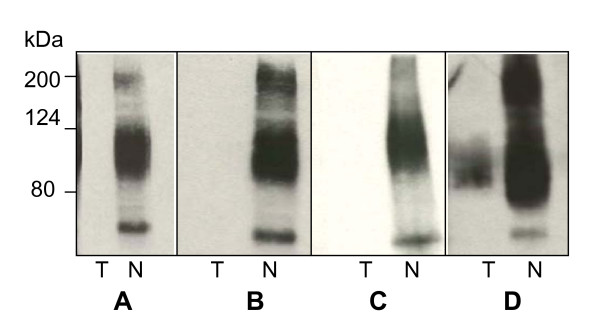
**Immunoblot analysis of gastric NIS expression in human gastric tumors (T) and normal peritumoral tissues (N)**. A, B, and D: adenocarcinoma, C: MALT lymphoma.

Immunohistochemical analysis of NIS in the thyroid, salivary glands, and stomach revealed very distinctly in which particular cells NIS is located in each tissue, namely the thyroid epithelial (Fig. 1C,D), salivary gland ductal epithelial (Fig. 1E,F), and gastric mucin-producing epithelial cells (Fig. 1G,H). In all three kinds of cells, NIS is clearly observed in the basolateral plasma membrane. By immunoblot analysis of membrane fractions, human NIS was detected mainly as a fully glycosylated mature ~90–120-kDa polypeptide in thyroid, salivary gland, and stomach (Fig. 1B and Fig. 2). We have previously shown that in rat tissues, NIS differences in electrophoretic mobility are due to different degrees of glycosylation[6]. The partially glycosylated ~50-kDa-precursor band and the ~180-kDa-dimer band observed in thyroid tissue were also detected in salivary glands and in the stomach upon longer exposure.

We investigated NIS protein expression by immnunoblot analysis in 17 gastric tumors (3 MALT and 14 adenocarcinomas) and in their corresponding peritumoral normal tissues. Twelve tumors (71%) exhibited no NIS expression and 5 (29%) of the tumoral tissues showed significantly decreased NIS expression compared to that of normal gastric mucosa (Table 1). Six peritumoral tissues displayed NIS expression similar to that of normal mucosa, 4 exhibited weak expression, and 7 lacked NIS expression altogether. No MALT lesions displayed NIS staining; interestingly, the normal mucosa in close proximity of the tumor was also negative (Table 1 and Fig. 2).

**Figure 3:**
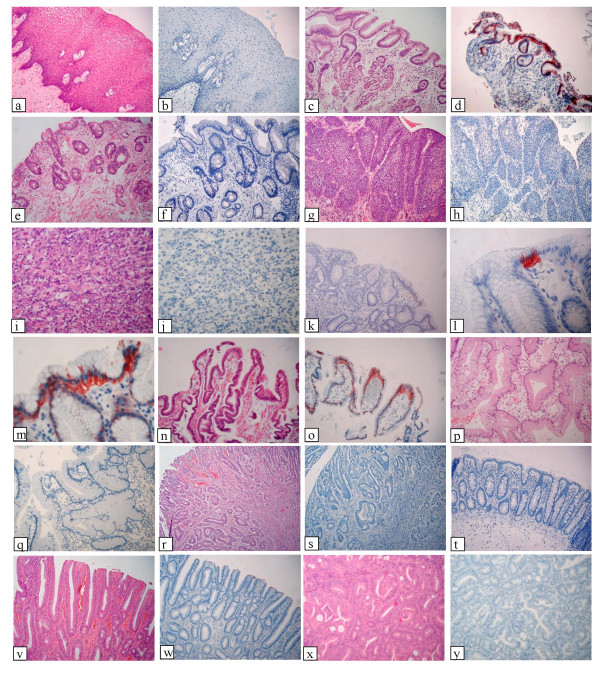
**Immunohistochemical analysis of NIS expression in human gastrointestinal tract**. **a**: normal esophageal squamous epithelium (haematoxylin-eosin/HE/staining, original magnification 100×) **b**: normal esophageal squamous epithelium: negative for NIS expression (original magnification 100×) **c**: Barrett mucosa (HE staining, original magnification 100×) **d**: Barrett mucosa, junctional and fundus-type columnar metaplasia: NIS positive staining (original magnification 100×) **e**: Barrett mucosa with intestinal metaplasia (HE staining, original magnification 100×) **f**: Barrett mucosa with intestinal metaplasia: negative for NIS expression (original magnification 100×) **g**: squamous cell esophageal carcinoma (HE staining, original magnification 100×) **h**: squamous cell esophageal carcinoma: NIS negative staining (original magnification 100×) **i**: gastric carcinoma – signet ring cell – (HE staining, original magnification 200×) **j**: gastric carcinoma – signet ring cell – negative for NIS expression (original magnification 200×) **k**: on the border of the gastric adenocarcinoma, adjacent "normal" extratumoral mucosa: faint, focal NIS expression (original magnification 100×) **I**: at 1 cm from gastric adenocarcinoma: definite focal NIS staining (original magnification 400×) **m**: far from the gastric adenocarcinoma: strong, linear NIS expression (original magnification 400×) **n**: gastric polyp (HE staining, original magnification 100×) **o**: gastric polyp: NIS positive staining (original magnification 100×) **p**: gastric polyp (HE staining, original magnification 200×) **q**: gastric polyp with colon-type metaplasia: NIS negative staining (original magnification 200×) **r**: small bowel adenocarcinoma (HE staining, original magnification 100×) **s**: small bowel adenocarcinoma: NIS negative staining (original magnification 100×) **t**: normal large bowel mucosa: NIS negative staining (original magnification 100×) **v**: colon polyp (HE staining, original magnification 100×) **w**: colon polyp: NIS negative expression (original magnification 100×) **x**: adenocarcinoma of the colon (HE staining, original magnification 100×) **y**: adenocarcinoma of the colon: negative for NIS expression (original magnification 100×).

To gain further insight into NIS expression in the digestive tract, we analyzed NIS by immunohistochemistry in samples from different patients. A total of 155 tissue samples obtained from the gastrointestinal tracts of 66 patients (Table 2) was studied, including 42 esophageal, 53 gastric, 11 small-bowel, and 49 large-bowel tissue samples. NIS expression was detected only in the gastric mucosa. More specifically, we observed NIS expression in the basolateral region of normal gastric mucosal surface epithelial cells in the form of expressed, homogenous linear staining (Figs. 1G,H; and 3m). At the same time, we detected no NIS expression in the mucin-containing cells of the neck of the foveola, in the parietal and chief cells, or in the neuroendocrine cells. Similarly, the goblet, Paneth, and ciliated epithelial cells of gastric intestinal metaplasia, as well as the mucus-producing columnar cells of the colon and the squamous esophageal epithelium, proved NIS negative (Fig. 3b,q,t,w).

NIS expression was absent in gastric cancer, independently of its histological type [adenocarcinoma (n = 4), signet-ring cell (n = 3), papillary (n = 1) and (Fig. 3i–q)]. NIS expression was undetectable along the tumor margins in more than half the cases and weakly focally present in the remaining instances (Table 3). Even at a distance of 1 cm from the tumor margin, NIS staining was weak and focal in all cases in contrast to that in normal gastric mucosa (Figs. 2G,H and 3k,I). The characteristic linear plasma membrane expression pattern was detected only far from the tumor (Fig. 3m), indicating that NIS expression is lost in the vicinity of the cancer.

Nine out often hyperplastic gastric polyps were NIS-positive (Fig. 3n,o), the only negative being a colon-type metaplasia (Fig. 3p,q). The findings in samples from patients with Barrett esophagus were striking: five junctional and fundus-type columnar metaplasias were NIS positive (Fig. 3c,d), whereas five showing intestinal metaplasia were consistently negative (Fig. 3e,f).

The samples from patients with gastritis displayed NIS expression independently of the presence (4/6) or absence (2/6) of *Helicobacter pylori *(Table 2).

## Discussion

The field of extrathyroidal I^- ^transport has changed considerably since the extensive review published on the topic in 1961 by Brown-Grant. The main vertebrate nonthyroid tissues reported to accumulate I^- ^actively via NIS are the salivary glands, gastric mucosa, lactating mammary gland, placenta, choroid plexus, and ciliary body of the eye[3,4,8,24]. With the exception of the lactating mammary gland, the physiological role of I^- ^or other anions transported by NIS in extrathyroidal tissues is unknown. These tissue-specific NIS-mediated transport systems exhibit functional similarities to their thyroid counterpart, such as a Km for I^- ^ranging from 10 to 30 μM and susceptibility to inhibition by perchlorate (CIO_4 _^-^). In contrast to TSH-regulated thyroid NIS and lactationally regulated mammary gland NIS, both salivary and gastric NIS are constitutively expressed[6,25,26]. NIS has been detected in the basolateral membrane of all ductal epithelial cells in the salivary gland[6], and in the basolateral membrane of superficial mucin-secreting epithelial cells in the stomach[6,13,15,27]. Although Spitzweg *et al *[27] reported NIS-specific immunostaining in parietal cells, we observed NIS expression exclusively in surface epithelial cells. Wapnir *et al *[14] have reported some NIS expression in a limited number of colon samples, but no NIS expression in colon was detected in the present study by either immunohistochemistry or immunoblot (not shown). There are at least three possible explanations for this discrepancy: NIS expression may be focal rather than widespread along the large bowel, variations in fixation methods may alter tissue antigenicity, or microarray overstaining may result from the "edge effect" of the small tissue cores.

NIS transports other anions with the following relative apparent affinities: I^- ^(1.00) ≥ SeCN^- ^(0.87) > SCN^- ^(0.34) > CIO_3 _^-^(0.12) > NO_3 _^- ^(0.04)[28]. As indicated earlier, the roles of I^- ^or other anions secreted to the lumen of the gastrointestinal system by the constitutively expressed salivary and gastric NIS are unknown. I^- ^concentrated in the gastric juice is reabsorbed in the small intestine and used for thyroid hormone biosynthesis by the thyroid or excreted by glomerular filtration through the kidney. Albeit with a lower affinity than I^-^, nitrate (NO_3 _^-^) is one of the other anions transported by NIS. Nitrate can be reduced to nitrite (NO_2 _^-^) by facultative anaerobic bacteria, and it can be acidified in the stomach, generating nitric oxide (NO), which has a strong bactericidal effect[29,30]. Indeed, acidified NO_2 _^- ^is bactericidal against *H. pylori*[31]; this effect is amplified by thiocyanate (SCN^-^), another NIS substrate[32]. *H. pylori *is frequently found in patients with gastric ulcers, suggesting a possible causative role for this bacteria[33]. Thus, one may speculate that if NIS-mediated NO_3 _^- ^secretion occurs in the stomach, NIS function might play a role in containing or preventing *H. pylori*-linked gastric ulcers or chronic gastritis. However, we observed no changes in NIS expression in samples from patients with gastritis as compared to normal subjects, irrespective of whether *H. pylori *was present.

NIS expression and function have been investigated in both thyroid and breast cancers. Whereas NIS expression is absent or decreased in 30% of thyroid cancers, NIS is actually overexpressed but not properly targeted to the plasma membrane in the remaining 70%[14,22]. Significantly, over 70% of human breast cancers express NIS, raising the prospect of the possible use of radioiodide in the diagnosis and treatment of breast cancer[6], as is routinely and successfully done in the treatment of NIS-expressing thyroid cancer.

We ascertained NIS expression in gastrointestinal tumors by carrying out immunohistochemical analysis on biopsies obtained from 66 patients, partly during surgical interventions and partly during endoscopical examinations. In addition, 17 surgical samples were investigated by immunoblot analysis. We detected no NIS staining in patients with intestinalization or gastric cancer, indicating that malignant transformation is linked to decreased or suppressed NIS expression. Interestingly, the focal NIS staining observed in the direct vicinity of gastric tumors increased gradually and became linear as we proceeded away from the tumor. This suggests that loss of NIS expression may precede microscopically identifiable morphological changes. One could speculate that NIS expression in the apparently normal gastric mucosa could be used to define the true healthy margin, thus becoming a potentially useful indicator to decrease local/anastomotic recurrences.

In junctional and fundus-type columnar metaplasia Barrett mucosa, NIS expression was intact; in contrast, in Barret mucosa with intestinalization, NIS expression was absent. A similar phenomenon was observed in hyperplastic gastric polyps with intestinal metaplasia. These data provide the molecular explanation for the ^99m^TcO_4 _^-^scintigraphic results from 30 years ago[16-18,20,21].

## Conclusion

Taken together, our findings underscore the prognostic and diagnostic significance of the absence of NIS expression in gastric alterations when intestinalization or cancer occurs. This is especially true in Barrett metaplasia, since the junctional and fundus-type metaplasias, in which NIS is normally expressed, pose a much lower risk of malignant transformation than intestinal metaplasia, which exhibits no NIS expression and gives rise to a higher number of dysplastic alterations and adenocarcinomas even in small (i.e., <3-cm) lesions[34]. In conclusion, this research suggests that the introduction of NIS immunohistochemical tests in gastric mucosa samples may be of considerable diagnostic value in Barrett esophageal and gastric polyps to evaluate intestinal metaplasia, and as an additional early molecular marker in the diagnosis of precancerous or/and cancerous gastroesophageal lesions.

## Competing interests

The author(s) declare that they have no competing interests.

## Authors' contributions

OD and NC conceived the study, AA, OD and NC designed the study and drafted the manuscript, OD and NC generated the NIS Abs and established the protocol for NIS immunohistochemistry, AA and ASZ collected the samples and carried out immunohistochemistries, OD and MP performed immunoblots, IW performed pertechnetate imaging. All authors read and approved the final manuscript.

## Pre-publication history

The pre-publication history for this paper can be accessed here:



## Figures and Tables

**Table 1:** Immunoblot analysis of Na^+^/l^- ^symporter protein expression in gastric tumors

**Case N°**	**Histology**	**Gender**	**Age**	**Immunoblot tumor**	**Immunoblot normal mucosa**
1	adenocarcinoma	M	71	-	-
2	MALT lymphoma	F	65	-	-
3	MALT lymphoma	F	70	-	-
5	adenocarcinoma	F	54	-	-
7	adenocarcinoma	F	30	-	+
8	adenocarcinoma	M	71	-	-
9	adenocarcinoma	M	69	-	+
10	MALT lymphoma	F	68	-	+++
11	adenocarcinoma	M	71	+	+++
12	adenocarcinoma	M	74	+	+++
13	adenocarcinoma	M	61	-	-
19	adenocarcinoma	M	46	+	+
20	adenocarcinoma	M	50	+	+++
27	adenocarcinoma	M	66	-	+
28	adenocarcinoma	M	68	-	+++
29	adenocarcinoma	M	61	-	+++
35	adenocarcinoma	M	62	+	-

**Table 2:** NIS expression in normal and diseased human gastro-intestinal tissues

	**Number of samples**	**NIS (+)**	**NIS (-)**
**Esophagus**	**42**		
- normal mucosa	4	0 (0 %)	4 (100 %)
-squamous cell carcinoma*	6	0 (0 %)	6 (100 %)
-adenocarcinoma	4	0 (0 %)	4 (100 %)
***- Barrett ***– junctional/fundus type	***5***	***5 (100 %)***	0 (0 %)
***- Barrett ***– intestinal metaplasia	***5***	0 (0 %)	***5 (100 %)***
**Stomach**	**53**		
***- normal***	***8***	***8 (100 %)***	0 (0 %)
-adenocarcinoma*	8	0 (0 %)	8 (100 %)
***-polyp (hyperplastic)***	***10***	***9 (90 %)***	1 (10 %)
***-gastritis***	***6***	***6 (100 %)***	0 (0 %)
**Small intestine**	**11**		
-normal	4	0 (0 %)	4 (100 %)
-inflammation	3	0 (0 %)	3 (100 %)
-polyp	2	0 (0 %)	2 (100 %)
-adenocarcinoma	2	0 (0 %)	2 (100 %)
**Colon**	**49**		
-normal	8	0 (0 %)	8 (100 %)
-inflammation	3	0 (0 %)	3 (100 %)
-polyp	9	0 (0 %)	9 (100 %)
-adenocarcinoma*	8	0 (0 %)	8 (100 %)

* Samples obtained from the corresponding peritumoral tissues are shown in Table 3; however, they are included in the total number of samples in this table.

**Table 3:** NIS expression in gastrointestinal tumors and adjacent tissues

**Localization of the samples**	**Number of samples**	**NIS (+)**			**NIS (-)**
		
		**linear expression**	**focal expression**		
				
			**distinct**	**faint**	
ESOPHAGUS					
Tumor	6	-	-	-	6
Border	6	-	-	-	6
1 cm distance	6	-	-	-	6
≥ 3 cm distance	6	3*	1*	-	2
STOMACH					
Tumor	7	-	-	-	7
Border	7	-	1	2	4
1 cm distance	7	-	2	5	-
≥ 3 cm distance	7	1	4	2	-
COLON					
Tumor	7	-	-	-	7
Border	7	-	-	-	7
1 cm distance	7	-	-	-	7
≥ 3 cm distance	7	-	-	-	7

(*gastro-esophageal junction)
